# Editorial: Sexual Behavior as a Model for the Study of Motivational Drive and Related Behaviors

**DOI:** 10.3389/fnbeh.2020.00121

**Published:** 2020-09-04

**Authors:** Fabrizio Sanna, Patrizia Porcu, Liana Fattore

**Affiliations:** ^1^Section of Neuroscience and Clinical Pharmacology, Department of Biomedical Sciences, University of Cagliari, Cagliari, Italy; ^2^CNR Neuroscience Institute, National Research Council, Cagliari, Italy

**Keywords:** sexual behavior, sexual motivation, mesocorticolimbic system, dopamine, sex hormones, motivational drive, animal models, individual differences

Sex is a strong, primary natural reinforcer with a pervasive role in the life of animals and human beings. However, sexual motivation differs from other classes of motivation, i.e., thirst and hunger, being necessary for the preservation of species but not for the survival of individuals. The study of the neurobiological underpinnings of sexual behavior may provide useful information about the mechanisms underlying its motivational determinants and, in more general terms, about the neural correlates of motivation, whose processes are dysfunctional in many psychopathological conditions, including major depression and addiction. Sexual behavior and its motivational determinants could therefore serve as a general model for investigating physiologically and pathologically motivated behaviors with the ultimate goal of identifying new therapeutic approaches for the treatment of psychogenic sexual dysfunctions and psychopathological conditions characterized by altered motivation and related dysfunctional behaviors.

Sexual behavior, and specifically sexual motivation, has traditionally been “hard matter” for the scientist who attempts to investigate it. Difficulties are immediately present in its definition, methodological approach and data interpretation. This Research Topic brings together 18 contributions (11 Original researches, four Reviews, two Mini-reviews, and one Brief Research Report) where comprehensive overviews of current knowledge on the neurobiology of sexual behavior and original findings on innovative approaches and models for studying sexual motivation in males and females are provided by leading experts in the field.

Firstly, a key point in the comparative research on sexual behavior and motivation, i.e., the generalization from animals to humans, is discussed by Le Moëne and Ågmo. Authors report that while huge behavioral differences exist between humans and rodents in terms of consummatory aspects, analogies can be observed, to some extent, in the motivational determinants that allow sexual interaction and copulation as well as in the underlying hormonal and neurochemical correlates. However, unlike in animals, the determinants of sexuality in humans are strongly influenced by social factors. The use of animal models of sexual behavior to investigate the features of human pathological conditions is elegantly discussed by Bialy et al.. Based on the analysis of the parameters that describe the complex structure of sexual behavior in laboratory rodents, the authors propose an interesting approach for delineating the distinct mechanisms affecting sexual motivation and performance in several (psycho)pathological conditions and assessing the efficacy of therapeutic approaches in preclinical investigations. To better characterize the multiple facets and complexities of sexuality, Portillo and Paredes discuss the broad spectrum of reproductive strategies in mammals in which biological variability points to the importance of understanding their neurobiological bases in different species and provide a description of asexuality and monogamous bonds in mammals—two conditions that can model and help us to understand some important aspects of human sexual motivation.

Employing selected rat lines is a relatively new strategy for the study of sexual behavior that allows investigation of the genetic and neurobiological underpinnings of individual differences. Esquivel-Franco et al. investigate the role of 5-HT1A auto- and hetero-receptors in sexual behavior in wildtype and knockout rats lacking the serotonin transporter (SERT), which model the alterations in ejaculatory function observed under conditions of chronically elevated levels of serotonin, e.g., during SSRI therapy. A transgenic animal model is also used by Sanna et al. who demonstrate altered motivational and performance aspects of sexual behavior in rats partially or totally lacking the dopamine transporter (DAT). Behavioral alterations were accompanied by an imbalanced dopamine/glutamic acid interaction and an altered expression of neural activation and plasticity markers in mesocorticolimbic areas, suggesting a possible use of these animals as a model of hypersexuality due to chronic hyperdopaminergia. The respective roles of genetic background and maternal care in affecting sexual responses are investigated by Dorantes-Nieto et al. in rat lines selected for differences in their yawning response. Authors observe that while some behavioral features/responses are resilient to environmental factors, thus revealing the strong influence of the genetic background, others can be modified by selective fostering, showing that maternal care is capable of changing innate behavioral responses, including yawning, penile erection and grooming.

Several contributions identify a key component of the neurobiological core of sexual motivation and drive in the dopamine mesocorticolimbic system. However, beyond the well-established involvement of this system in motivational processes, what emerges is the need for broader research to better understand the complex neurochemical interactions between dopamine and other neurotransmitters in modulating its activity. Moore et al. employ biosensors and DREADD technique to investigate fronto-accumbal glutamatergic activity during sexual behavior in the female hamster. What emerges from this study is the importance of the fronto-cortical glutamatergic input to mesolimbic dopaminergic areas in fine modulation of the behavioral output, mainly regarding its motivational aspects, which provides new insight into the neurobiology of the motivational control of female sexual behavior. Canseco-Alba and Rodríguez-Manzo examine the interaction between mesolimbic dopamine and endocannabinoids in regulating sexual motivation and satiation in the male rat. They show that endocannabinoid activity reverses sexual satiety by modulating dopaminergic transmission, presumably at the mesolimbic system, with anandamide and 2-arachidonoylglycerol, displaying different actions on D1- and D2-like receptors.

Sexual dysfunction in women is poorly understood, perhaps due to its subtle expression; this can include loss of motivation and loss of pleasure during sex, which can affect intimate relationships, self-esteem and, ultimately, psychological well-being and quality of life. Animal models of female sexual behavior have provided insight on how the interaction of neurotransmitters and steroid hormones are required to modulate the central motivation state and thus affect sexual motivation. Some of the contributions to this Research Topic focus on the neurobiological mechanisms involved in female sexual motivation at both preclinical and clinical levels. Guarraci and Frohardt review the current knowledge on models of sexual motivation in female rats and discuss the main patterns of behavior that reflect either increases or decreases in motivation to advance our understanding of female sexual behavior with particular attention to partner choice and preference models. The role of sex hormones in modulating female sexual motivation when multiple rewards (e.g., food and sex) are available is examined by Yoest et al.. They provide a neurobiological framework for understanding how ovarian hormones, released over the course of the estrous cycle, modulate adaptive behavioral choices in females when multiple rewards are available.

The role of sex hormones in modulating pathologically motivated behavior is investigated by Bakhti-Suroosh et al. who examine how estradiol affects different aspects of psychostimulant addiction. Notably, a dual role for this steroid hormone in drug addiction was revealed. Estradiol was found to both enhance and reduce vulnerability by amplifying drug reward and facilitating new learning during the extinction process, respectively. The interactions between psychostimulants, ovarian hormones and sexual motivation are reviewed by Rudzinskas et al. who discuss a model of increased sexually-motivated behaviors induced by administration of the psychostimulant methamphetamine in females. They suggest that the combination of ovarian hormones, olfactory information and methamphetamines could produce enhanced sexual motivation by inducing activation and neural plasticity within a key integration site for sexually relevant sensory information, i.e., the posterodorsal medial nucleus of the amygdala.

Besides sex hormones, the neurosteroid allopregnanolone is now attracting the attention of researchers for its ability to modulate specific aspects of female sexuality. In their first contribution, Frye et al. show that the effects of intra-VTA allopregnanolone on female sexual behavior involve NMDA receptors and are likely mediated through GABA-A receptors. A second contribution of Frye and Chittur investigates the neuroplastic modifications induced by mating in the mesocorticolimbic system of female rats and shows that mating significantly enhances midbrain mRNA expression of genes involved in hormonal and trophic actions, revealing a complex fine-tuning of mating-induced neuroplastic processes.

Preclinical research on sexual behavior has almost exclusively been conducted in mammals, mainly rats. Here, Sato et al. review the role of Fru proteins in the sexual behavior of *Drosophila melanogaster* and discuss how the selective expression of some forms of these proteins might confer male-specific roles by interacting at transcriptional level with partner proteins, thus contributing to the regulation of male sexual behavior with particular reference to courtship and mating.

Three clinical studies conclude the Research Topic. In the first study, Regier et al. investigate possible differences in the activity of the mesolimbic system in women with different propensities of engaging in unsafe sexual intercourse. Authors report that women who have protected sex may view sexually related stimuli more positively than young women at increased risk of sexually transmitted diseases (STIs)/HIV, in which they also evidenced lower mesolimbic responses to sexual cues. The study thus enriches the list of prevention factors and may help to identify young women at greatest risk of contracting STIs and/or HIV. The large multicenter study by Zamboni et al. focuses on sexual functioning in opioid-addicted women under opioid maintenance treatment and reveals that the majority of them have sexual dysfunction, regardless of the treatment protocol (buprenorphine vs. methadone), and report a poor quality in intimate relationships and mental health. These results suggest that female sexual well-being should also be taken into account during treatment detoxification given that it may impact adherence to therapy and, thus, interfere with its beneficial outcomes. Finally, Soares et al. investigate the association between infatuation/passionate love and impulsivity in adolescents with Attention Deficit and Hyperactivity/impulsivity Disorder (ADHD). Intriguingly, although an association between infatuation intensity, behavioral urgency, and sensation-seeking was observed, this association does not change in the presence of ADHD, pointing to the need for further studies to clarify an increased risk for negative social outcomes due to sexually related risky behaviors in some population groups.

In conclusion, the classical Beach's distinction between appetitive and consummatory aspects of sexual behavior seems to be, in its core aspects, still valid and constitutes a conceptual framework for most of the recent research in the field. However, current challenges lie in depicting the extremely complex intricacies between these two main aspects of sexual behavior, accounting for its complexity at both behavioral and neurobiological levels. The disclosure of the neurobiological underpinnings of sexual behavior at molecular, neuronal, and system levels is dramatically improving our knowledge of this complex matter. The studies on the specific roles of different neurosteroids and their interactions with brain neurotransmitters as well as on the neuroplastic changes induced by sexual activity will be highly informative in accounting for such a complexity. Genetic and (bio)behavioral selection have allowed for a more accurate investigation of the determinants and neurobiological correlates of individual differences related to sexual motivation and behavior. Significant strides have been made in the study of difference between the sexes, and the increasing number of studies on female sexuality is important to note, as research on female sexual behavior has often been “neglected.”

Overall, we feel that the present Research Topic provides an interesting and valuable picture of the current field and contributes to our understanding of the mechanisms that control sexual motivation and reward.

## Author Contributions

FS, PP, and LF equally contributed to this Editorial for the Research Topic entitled “Sexual Behavior as a Model for the Study of Motivational Drive and Related Behaviors.” All authors contributed to the article and approved the submitted version.

## Conflict of Interest

The authors declare that the research was conducted in the absence of any commercial or financial relationships that could be construed as a potential conflict of interest.

